# Plasmablastic Lymphoma Manifesting As Pleural Thickening and Effusion: A Case Report

**DOI:** 10.7759/cureus.73961

**Published:** 2024-11-18

**Authors:** Mohammed Elsayed, Abdelrahman Ali, Giedre Andrijevskiene

**Affiliations:** 1 Medicine, The James Cook University Hospital, Middlesbrough, GBR; 2 Medicine and Surgery, The James Cook University Hospital, Middlesbrough, GBR; 3 Radiology, The James Cook University Hospital, Middlesbrough, GBR

**Keywords:** extraoral plasmablastic lymphoma, hiv, oncology, pleural effusion, pleural thickening, radiology

## Abstract

Plasmablastic lymphoma (PbL) is a subtype of diffuse large B-cell lymphoma, primarily linked to human immunodeficiency virus (HIV) infection. This case report presents a 34-year-old HIV-positive patient who exhibited unusual signs of pleural thickening and effusion. Initial evaluations, including imaging and pleural fluid analysis, suggested thoracic empyema. However, histopathological examination ultimately revealed a diagnosis of PbL. Cyclophosphamide, doxorubicin, vincristine, and prednisolone (CHOP) chemotherapy were initiated, but the patient passed away within a few months. This case highlights the complexity of diagnosing PBL and its poor prognosis, particularly in immunocompromised individuals, stressing the importance of early detection and intervention.

## Introduction

Plasmablastic lymphoma (PbL) is a rare and aggressive subtype of diffuse large B-cell lymphoma, well-known for its association with human immunodeficiency virus (HIV) positive patients [1]. While PBL typically affects the oral cavity or gastrointestinal tract [2], rare cases have been documented presenting with pulmonary involvement [3], manifesting as pleural thickening and effusion.

PBL presenting with pleural thickening and effusion is particularly challenging to diagnose due to its overlapping clinical and radiological features with more common pleural pathologies, such as tuberculosis or malignant effusions. The definitive diagnosis relies on histopathological analysis [4], revealing plasmablasts with immunohistochemical profiles expressing plasma cell markers.

This case describes a patient with HIV who presented with pleural thickening and effusion, highlighting the importance of considering PbL in the differential diagnosis of pleural abnormalities, especially in immunocompromised patients, to ensure timely and appropriate treatment.

## Case presentation

**Figure 1:**
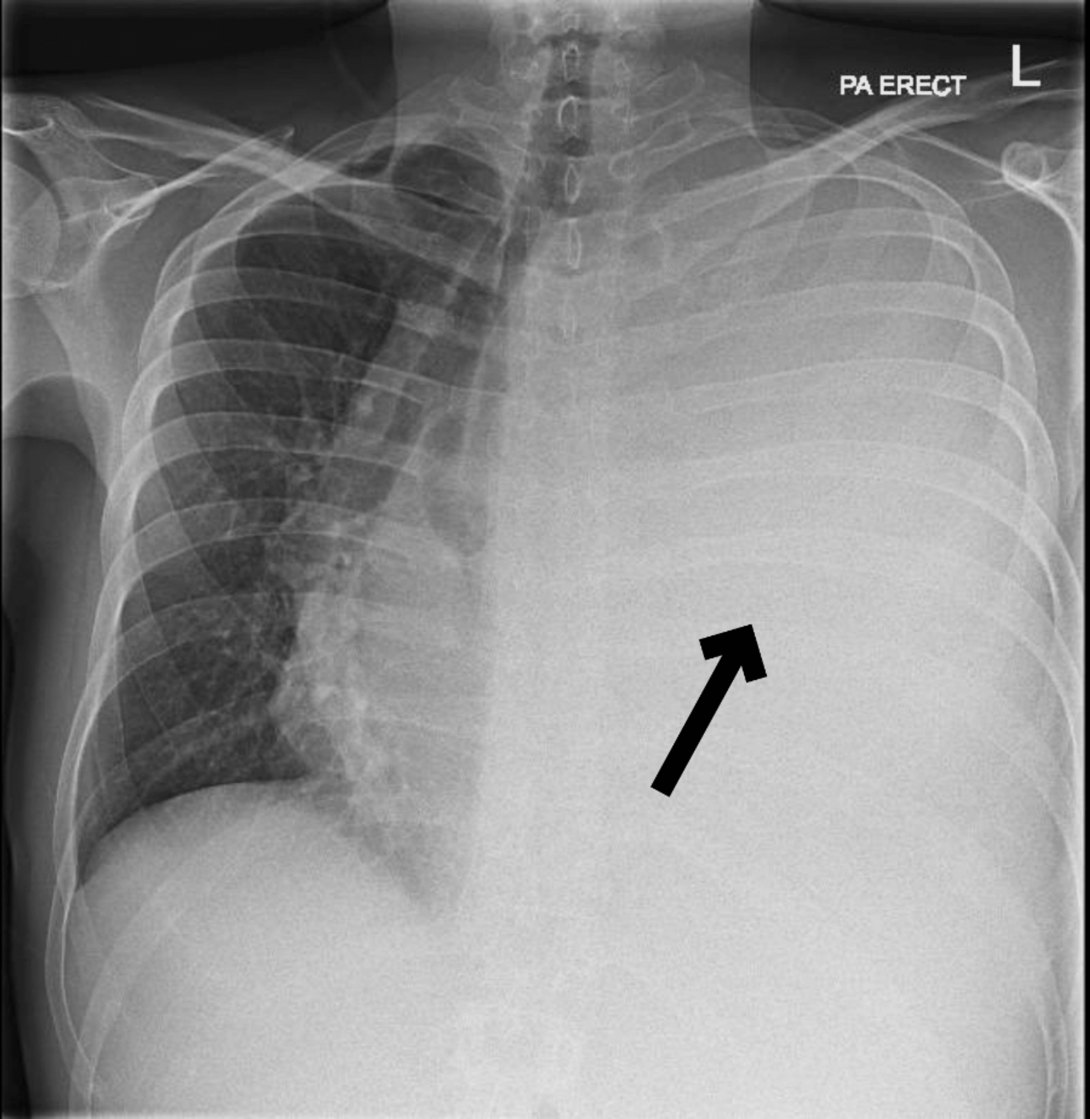
Chest x-ray (posterior to anterior) showing white out of left hemithorax with contralateral mediastinal shift suggesting large pleural effusion. Black arrow indicating pleural effusion

A 34-year-old male with a history of HIV, diagnosed in September 2023 and on anti-retroviral therapy, was admitted on November 11, 2023, complaining of constipation and pleuritic chest pain. Physical examination revealed decreased breath sounds and dull percussion on the left side of the chest. The rest of the examination was unremarkable. A chest x-ray (CXR) in the emergency department showed complete opacification of the left hemithorax, consistent with a large pleural effusion (Figure 1). A chest drain was inserted, draining 6 liters of blood-stained fluid. Pleural fluid studies showed lactate dehydrogenase (LDH) 1884 U/L, protein 45 g/L, with negative cultures and scant atypical lymphocytes present, glucose 6.5 mmol/L, and pH was not done.

**Figure 2:**
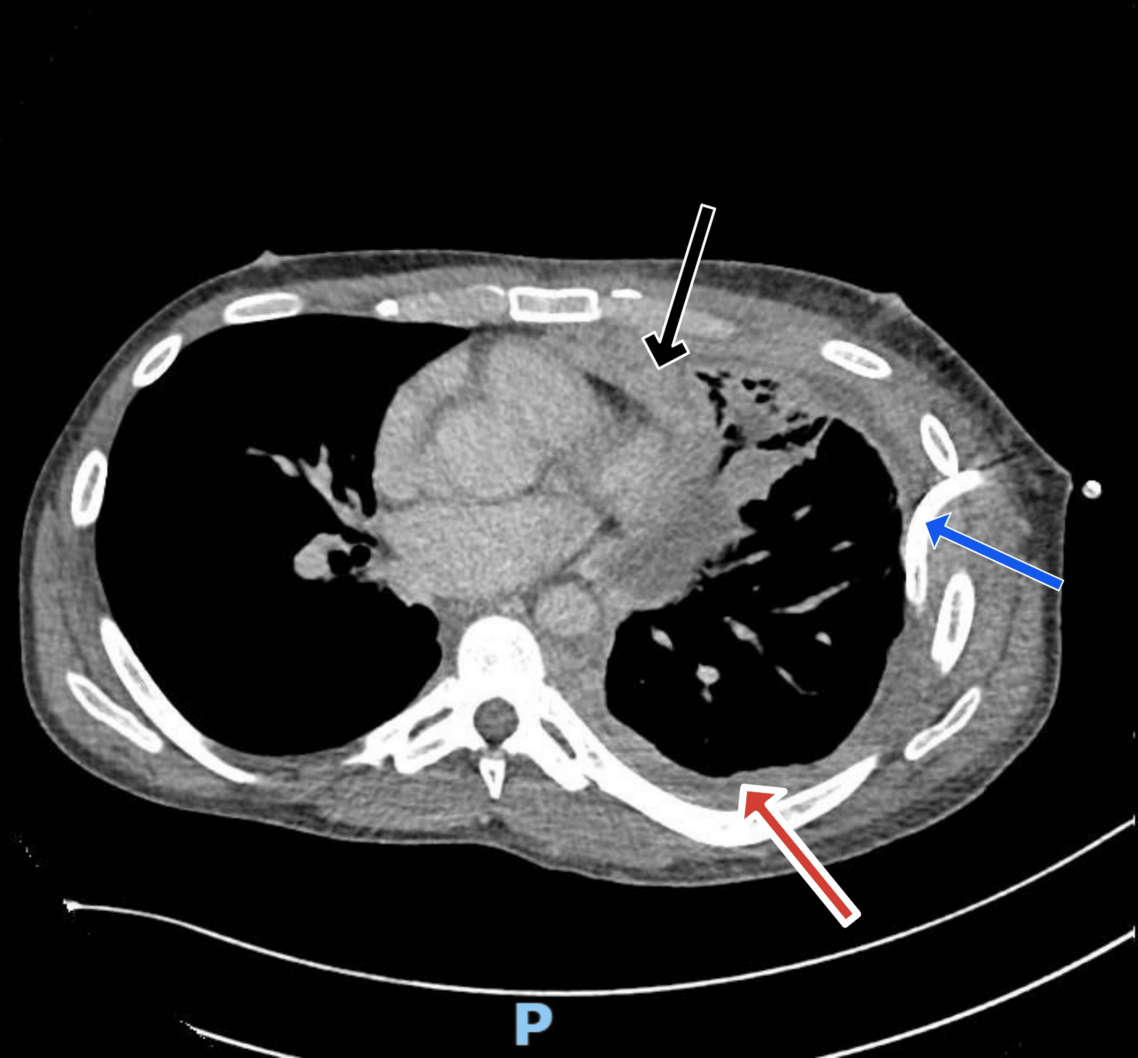
Axial CT chest image showing left-sided effusion with mild pleural thickening suggesting pleural empyema. Chest drain in situ. Black arrow indicating pleural thickening; Red arrow indicating pleural effusion; Blue arrow indicating chest drain

Routine blood tests are shown in Table 1. Blood cultures were negative. Computed tomography (CT) imaging on the same day suggested left-sided thoracic empyema and infection without firm evidence of underlying malignancy (Figure 2). Antibiotics were started after consulting with the microbiology team. The chest drain drained an additional 4 liters of fluid the following day.

**Table 1:** Laboratory investigations.

Laboratory test	Value	Normal range
White blood cell count	10.6 x10^9/L	(4.0 – 11) x10^9/L
Neutrophil count	8.0 x10^9/L	(2 - 7.5) x10^9/L
lymphocyte count	1.3 x10^9/L	(1.5 – 4) x10^9/L
Haemoglobin	143 g/L	130 – 180 g/L
Platelets	582 x10^9/L	(150 – 400) x10^9/L
C-reactive protein	100 mg/L	<5 mg/L
CD4 absolute level	151 cells/mm^3	410-1590 cells/mm^3

**Figure 3:**
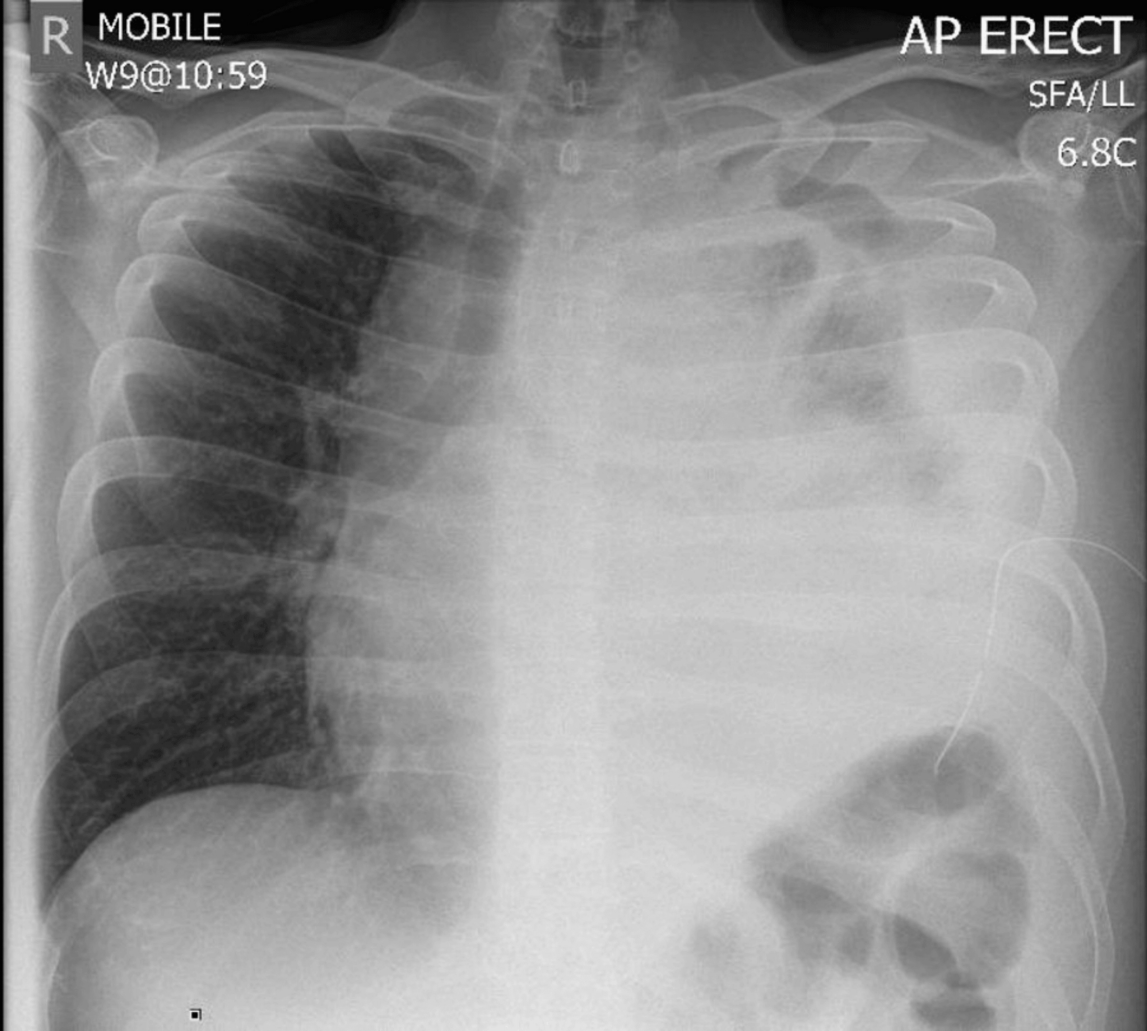
Chest x-ray showing persistent large left-sided pleural effusion.

**Figure 4:**
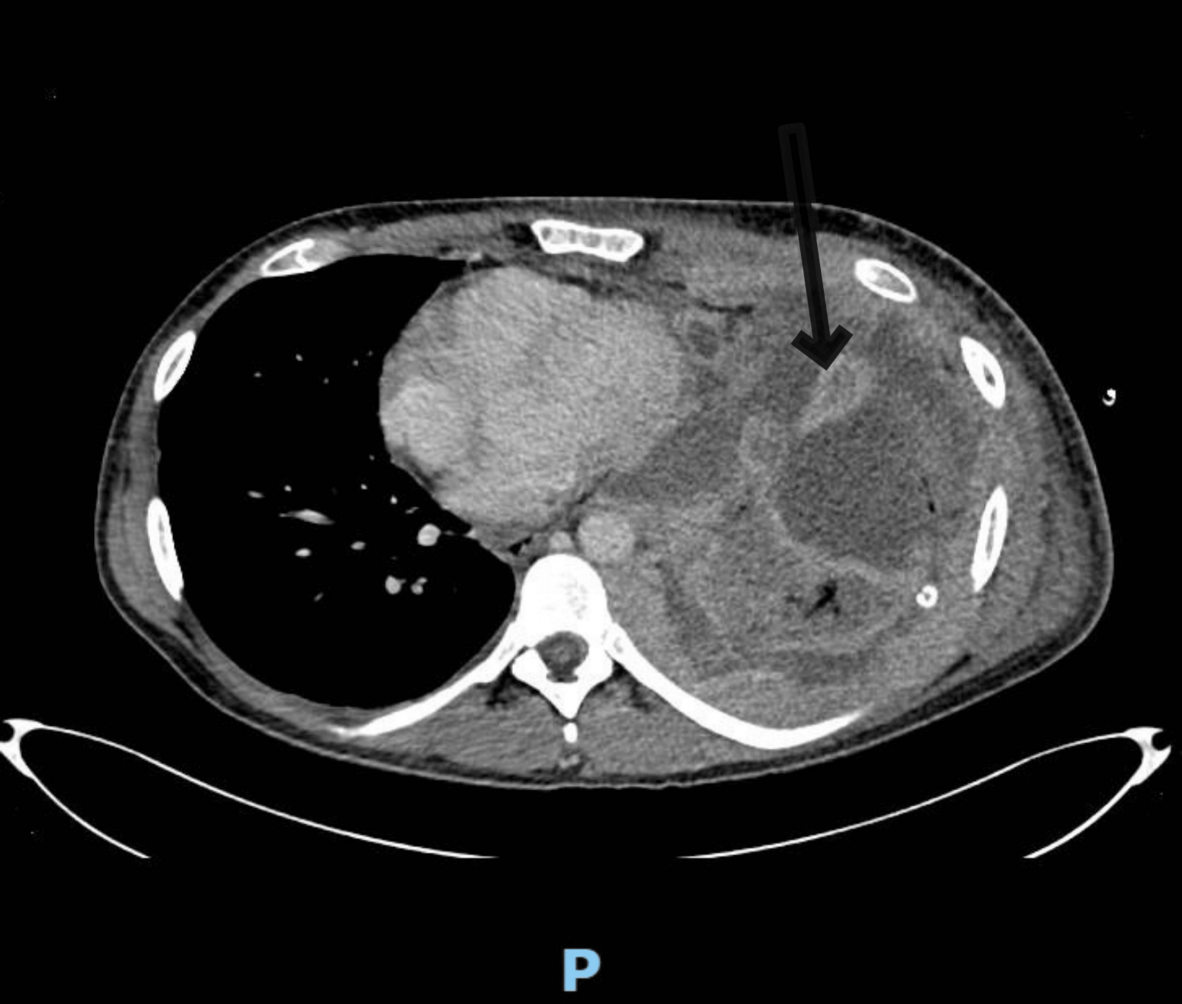
CT chest showing significantly increased left-sided nodular pleural thickening and effusion. Black arrow indicating nodular pleural thickening

On November 13, 2023, a repeat CXR (Figure 3) showed a persistent large left-sided pleural effusion. Advice was sought from the cardiothoracic surgery team regarding video-assisted thoracoscopic surgery (VATS) to look for cause and treat which they deemed the patient not for surgical intervention, to monitor progress, and to request another CT of the chest with contrast. The CT scan (Figure 4) revealed increasing consolidation throughout the left lung with nodular interlobular septal thickening in the left apex, new patchy opacities in the right middle and upper lobes, and extensive circumferential enhancing nodular pleural thickening with a slightly increased rightward mediastinal shift, a left superior mediastinal soft tissue mass encasing left subclavian artery, common carotid artery, and brachiocephalic vein is confluent with the pleural thickening in the left apex. The differential diagnosis suggested by the CT scan included primary pleural lymphoma, pyothorax-associated lymphoma, and mesothelioma. Pleural biopsy and fluid analysis were advised, and a lung multidisciplinary team (MDT) discussion was recommended.

**Figure 5:**
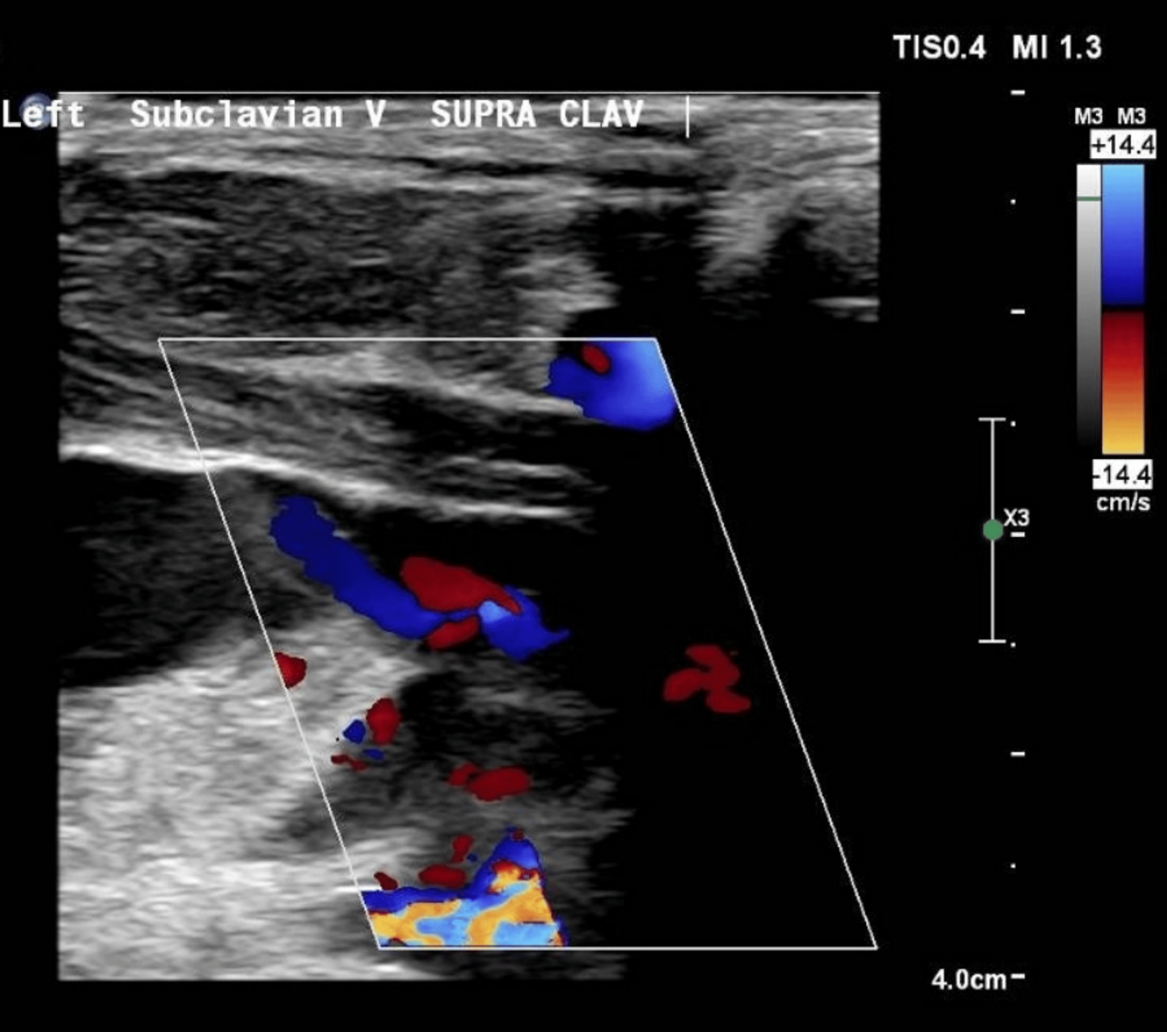
Doppler ultrasound of Left Supra-clavicular subclavian vein displays poor colour filling suggesting thrombus.

On November 14, the patient developed left arm swelling and cyanosis. A Doppler ultrasound (Figure 5) showed a left supra clavicular vein filling defect suggesting a thrombus. The patient was started on treatment-dose low molecular weight heparin. Despite antibiotics and oxygen, the patient’s condition deteriorated, requiring more oxygen supplementation. After discussions with cardiothoracic surgeons, the respiratory team, and radiologists, it was concluded that the patient was at high risk for complications of surgical washout and pleural biopsy, thus A CT-guided biopsy was planned.

**Figure 6:**
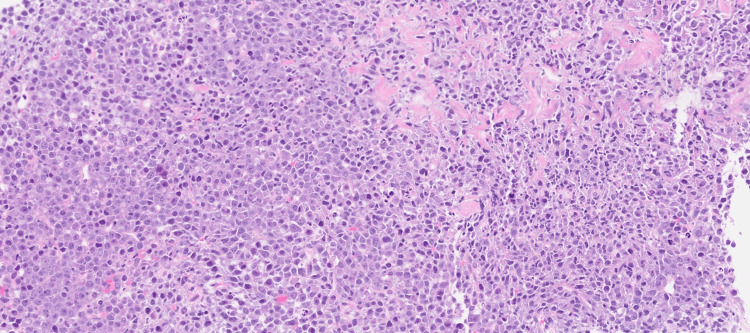
Specimen of pleural biopsy showing cores infiltrated by diffuse sheets of plasmacytoid cells with pleomorphic nuclei, prominent nucleoli with brisk mitotic activity and apoptotic bodies.

**Figure 7:**
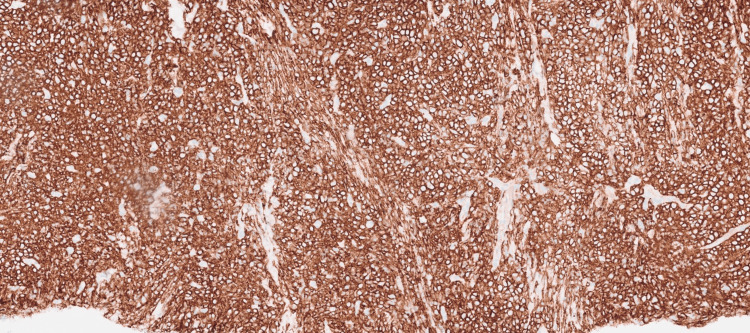
Immunohistochemical stain with anti-CD138 antibody.

**Figure 8:**
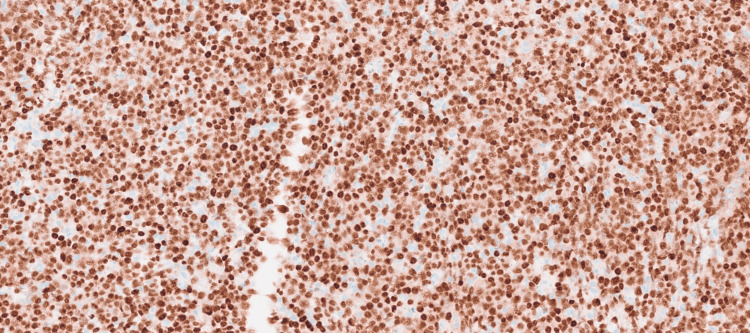
Immunohistochemical stain with anti-MUM1 antibody.

**Figure 9:**
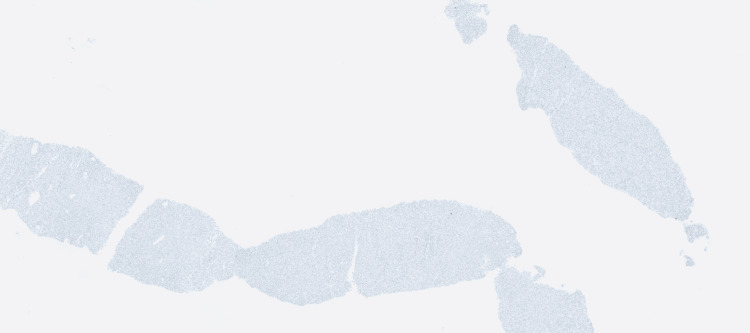
Immunohistochemical stain with anti-CD20 antibody.

**Figure 10:**
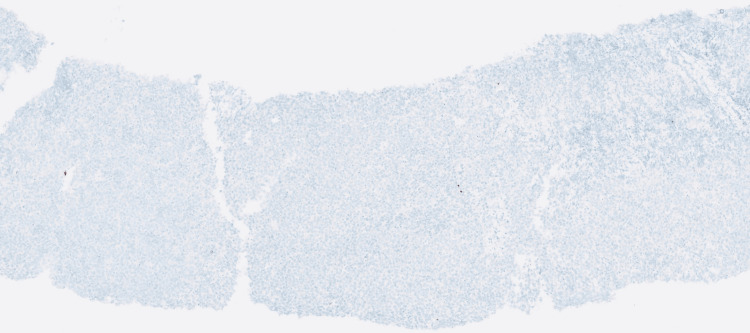
Immunohistochemical stain with anti-CD30 antibody.

**Figure 11:**
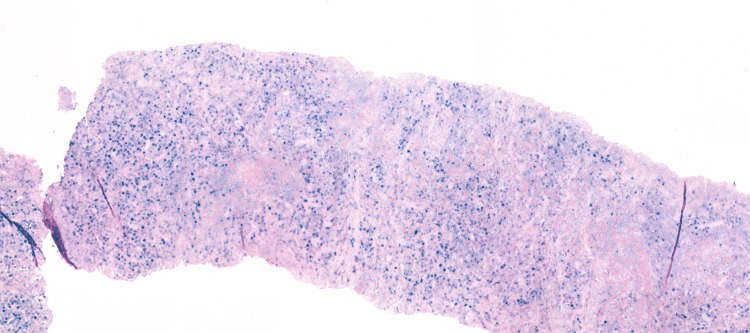
EBER in-situ hybridization. EBER: Epstein-Barr encoding region

**Figure 12:**
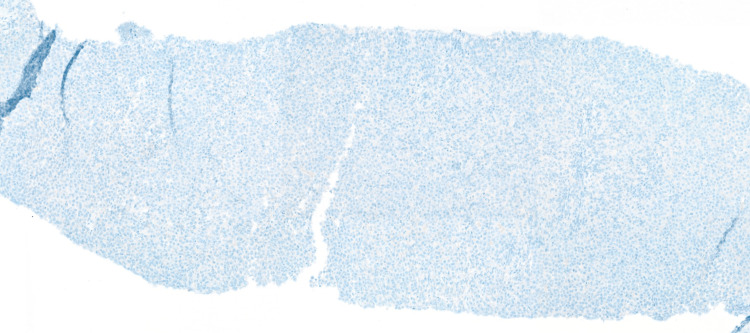
Immunohistochemical staining for HHV-8. HHV-8: Human Herpesvirus 8

Histopathological examination of the pleural biopsy revealed diffuse sheets of plasmacytoid cells with pleomorphic nuclei, prominent nucleoli with brisk mitotic activity, and apoptotic bodies (Figure 6). Immunohistochemistry showed positivity for cluster of differentiation (CD)45, CD138 (Figure 7), multiple myeloma oncogene 1 (MUM1) (Figure 8), CD10, and negativity for CD20 (Figure 9), CD30 (Figure 10), CD2, CD3, CD4, CD8, CD43, CD56, Epstein-Barr virus latent membrane protein 1 (EBV-LMP1), myeloperoxidase, SRY-Box transcription factor (SOX10), and TdT. The Ki-67 proliferation index was approximately 95%. Epstein-Barr encoding region (EBER) in-situ hybridization was positive (Figure 11) and human herpesvirus (HHV)-8 negative (Figure 12), confirming a diagnosis of plasmablastic lymphoma.

The patient was referred to the hematology team who deemed the patient at stage IV and started chemotherapy with cyclophosphamide, doxorubicin, vincristine, and prednisolone (CHOP). The patient responded to treatment and was discharged home with a hematology follow-up. On April 2024, the patient attended a hematology clinic with a three-day history of vomiting and very poor oral intake where he was admitted to the hospital but unfortunately died shortly after that.

## Discussion

Plasmablastic lymphoma (PbL) is an aggressive subtype of non-Hodgkin lymphoma characterized by the proliferation of immature lymphoid cells in the lymph nodes and extra-nodal sites. It affects a variety of locations, including the gastrointestinal tract, lymph nodes, skin, bones, genitourinary tract, nasal, and paranasal sinuses, although it is most frequently seen in the oral cavity [2,5,6].

PbL is classified into three distinct categories by Mani et al. [4]: PbL variant localized to the oral mucosa with possible nodal or extranodal sites, with minimal or no plasmacytic differentiation; PbL distinguished by plasmacytic differentiation and extraoral presentation; and PbL associated with HHV-8 and multicentric Castleman disease. While HIV/AIDS patients have a much higher incidence, immunocompetent persons have also been reported to have PBL [7].

PBL represents approximately 2% of HIV-associated lymphomas [8] with a median age of diagnosis in the fourth decade of life. Patients with PBL often have a poor prognosis, with a median overall survival of six to 19 months with no significant difference in prognosis between HIV-negative and HIV-positive patients as shown in a meta-analysis of 277 patients [9,10]

Pleural thickening and effusion in our patient as primary presentations, are exceedingly rare. This instance illustrates one aspect of the challenge in diagnosing this rare cancer suggesting that PbL may involve a wider range of anatomical sites than previously believed.

The Ann Arbor classification is used for staging PBL, with most patients falling into either stage I or IV [11]. The first and most used staging test for lymphomas is contrast-enhanced computed tomography (CECT) of the neck, abdomen, and thorax whilst according to Lugano 2014 criteria, Fluorodeoxyglucose positron emission tomography (FDG-PET) is the gold standard for evaluating response to chemotherapy in FDG-avid lymphomas like PBL [12,13].

The high clinical suspicion and early diagnosis of PBL are particularly critical given the high mortality rates and scarcity of research on effective treatment. High-quality randomized clinical trials examining the best course of treatment are few due to their rarity. However, due to its aggressive behavior, conventional CHOP therapy is deemed insufficient. Therefore, more intense regimens like dose-adjusted - etoposide, prednisone, vincristine, cyclophosphamide, and doxorubicin (DA-EPOCH), hyper-fractionated cyclophosphamide, vincristine, doxorubicin, dexamethasone, and high-dose methotrexate and cytarabine (Hyper-CVAD-MA), and cyclophosphamide, vincristine, doxorubicin, high-dose methotrexate/ifosfamide, etoposide, and high-dose cytarabine (CODOX-M/IVAC) are recommended [14-16].

## Conclusions

This case highlights the diagnostic and therapeutic challenges of plasmablastic lymphoma (PBL) manifesting as pleural thickening and effusion in an HIV-positive male. Despite initial interventions, the late-stage diagnosis and rapid progression underscored the aggressive nature of the disease. The unusual presentation delayed diagnosis, with imaging initially suggesting empyema rather than malignancy. Immunohistochemical confirmation of PBL allowed for chemotherapy initiation, but the patient ultimately succumbed to the disease. This case emphasizes the importance of considering PBL in immunocompromised patients with atypical pleural effusions and the limited prognosis even with treatment.
